# ¿Leche para todos?: Persistencia de las recomendaciones universales frente a la diversidad genética poblacional en Jujuy, Argentina

**DOI:** 10.18294/sc.2026.6035

**Published:** 2026-06-03

**Authors:** Madalena F. Monteban, Lorena Claudia García

**Affiliations:** 1 Doctora en Antropología. Investigadora asistente, Consejo Nacional de Investigaciones Científicas y Técnicas, Unidad Ejecutora en Ciencias Sociales Regionales y Humanidades, Universidad Nacional de Jujuy, San Salvador de Jujuy. Argentina. mmonteban@cisor.unju.edu.ar Universidad Nacional de Jujuy Consejo Nacional de Investigaciones Científicas y Técnicas Unidad Ejecutora en Ciencias Sociales Regionales y Humanidades Universidad Nacional de Jujuy San Salvador de Jujuy Argentina mmonteban@cisor.unju.edu.ar; 2 Licenciada en Antropología. Becaria doctoral, Consejo Nacional de Investigaciones Científicas y Técnicas, Unidad Ejecutora en Ciencias Sociales Regionales y Humanidades, Universidad Nacional de Jujuy. San Salvador de Jujuy, Argentina. lorenagarcia1625@gmail.com Universidad Nacional de Jujuy Consejo Nacional de Investigaciones Científicas y Técnicas Unidad Ejecutora en Ciencias Sociales Regionales y Humanidades Universidad Nacional de Jujuy San Salvador de Jujuy Argentina lorenagarcia1625@gmail.com

**Keywords:** Intolerancia a la Lactosa, Variación Genética, Programas y Políticas de Nutrición y Alimentación, Población Indígena, Guías Alimentarias, Argentina, Lactose Intolerance, Genetic Variation, Nutrition Programs and Policies, Indigenous Peoples, Food Guide, Argentina

## Abstract

Las guías alimentarias recomiendan 2-3 porciones diarias de lácteos de manera universal, sin considerar la variación genética poblacional regional. En la provincia de Jujuy, Argentina, la evidencia genética documenta entre el 53% y el 69% de ancestría indígena en la población general, sugiriendo alta prevalencia de hipolactasia primaria. Este ensayo analiza la desconexión entre las políticas nutricionales universales y la composición genética de poblaciones con alta ancestría indígena mediante cuatro ejes analíticos: 1) la hipolactasia como condición mayoritaria mundial (del 65% al 70% de la población global) y las mediaciones culturales en el consumo; 2) las trayectorias históricas de políticas lácteas en América Latina desde mediados del siglo XX, documentando cómo la investigación científica desde los años 1960 identificó prevalencias elevadas de hipolactasia en poblaciones latinoamericanas; 3) las prácticas alimentarias en instituciones de salud y educación de Jujuy, documentando que el rechazo sistemático de leche líquida se conceptualiza como fenómeno cultural-educativo más que fisiológico; y 4) el diseño de políticas públicas nutricionales culturalmente situadas. El análisis evidencia que medio siglo después de que la literatura científica identificara el fenómeno, las políticas argentinas continúan naturalizando el patrón metabólico minoritario europeo como norma universal. Se propone el desarrollo de un suplemento en las guías nacionales para Jujuy que reconozca explícitamente la diversidad genética poblacional y ofrezca múltiples vías para alcanzar requerimientos de calcio, siguiendo el modelo de otras intervenciones de salud pública argentina basadas en prevalencias poblacionales.

## Abstract (en)

Dietary guidelines universally recommend the consumption of 2-3 daily servings of dairy products, without considering regional population-level genetic variation. In the province of Jujuy, Argentina, genetic evidence documents between 53% and 69% Indigenous ancestry in the general population, suggesting a high prevalence of primary hypolactasia. This essay analyzes the disconnection between universal nutritional policies and the genetic composition of populations with high Indigenous ancestry through four analytical axes: 1) hypolactasia as the global majority condition (affecting 65%-70% of the world population) and the cultural mediations surrounding dairy consumption; 2) the historical trajectories of dairy policies in Latin America since the mid-twentieth century, documenting how scientific research since the 1960s identified high prevalences of hypolactasia in Latin American populations; 3) food practices within health and educational institutions in Jujuy, documenting how the systematic rejection of liquid milk is conceptualized as a cultural-educational rather than a physiological phenomenon; and 4) the design of culturally situated public nutritional policies. The analysis shows that, half a century after the scientific literature identified this phenomenon, Argentine policies continue to naturalize the minority European metabolic pattern as a universal norm. The article proposes the development of a supplement to the national dietary guidelines for Jujuy that explicitly acknowledges population genetic diversity and offers multiple pathways to meet calcium requirements, following the model of other Argentine public health interventions based on population prevalences.

## Introducción

### Historicidad del problema: políticas lácteas en América Latina desde la década de 1960

Desde mediados de la década de 1960, investigadores latinoamericanos comenzaron a documentar la alta prevalencia de hipolactasia en sus propias poblaciones. En Colombia, Alzate *et al*.^1^ describieron intolerancia a la lactosa en estudiantes de medicina y, al año siguiente, en adultos indígenas Chamí, señalando explícitamente sus implicancias socioeconómicas^2^. En Brasil, Vasconcellos y Gonçalves publicaron en 1968^3^ los primeros datos sobre deficiencia de lactasa en adultos. Paralelamente, en EEUU, Bayless y Rosensweig^4^ documentaban alta prevalencia de malabsorción de lactosa en población afroamericana, y Paige *et al*.^5^ extendían esa investigación a niños de países latinoamericanos. Este conjunto de evidencia fue sintetizado por Torún, Solomons y Viteri^6^ en una revisión de 194 publicaciones sobre América Latina publicada en los Archivos Latinoamericanos de Nutrición, en 1979, que constituyó la sistematización regional más exhaustiva hasta entonces.

Sin embargo, la evidencia acumulada no condujo a un cuestionamiento de los programas de distribución masiva de leche impulsados, en parte, por la Alianza para el Progreso -programa estadounidense lanzado en 1961-, sino a estrategias de adaptación que mantenían la leche como eje central de las intervenciones nutricionales. Medio siglo después, las Guías Alimentarias para la Población Argentina (GAPA) mantienen la recomendación universal de 2-3 porciones diarias de lácteos sin ajuste para contextos poblacionales donde la hipolactasia constituye la norma biológica mayoritaria^7^. En provincias como Jujuy, donde entre el 53% y el 69% de la población presenta ancestría genética indígena^8^, esta recomendación universal implica prescribir un consumo que la mayoría genética de la población no puede metabolizar eficientemente. Cabe señalar que Argentina carece de datos nacionales propios de persistencia de lactasa^9^, lo que significa que estas recomendaciones se implementan sin una base de prevalencia local verificada. Esta desconexión entre la evidencia acumulada durante más de cinco décadas y las recomendaciones vigentes es el punto de partida del análisis que desarrollamos en este trabajo.

### El problema de investigación: entre conocimiento científico y política alimentaria

Profesionales de la salud y la educación en Jujuy -agentes sanitarios, enfermeras, educadoras para la salud, nutricionistas, médicos, docentes y personal de programas alimentarios- reportan un patrón recurrente en instituciones escolares y de salud pública: niños y niñas que sistemáticamente rechazan el consumo de leche ofrecida con té o mate cocido en programas nutricionales institucionales. Este fenómeno, frecuentemente interpretado como una “aversión cultural” que debe superarse mediante educación alimentaria, se inscribe en la historia latinoamericana documentada desde la década de 1960, y persiste sin resolución en las políticas actuales.

Es importante reconocer desde el inicio el compromiso y esfuerzo sostenido de estos profesionales, quienes trabajan diariamente para mejorar la nutrición infantil en condiciones frecuentemente desafiantes, implementando programas que incluyen seguimiento nutricional sistemático, distribución de suplementos, educación alimentaria, y articulación con servicios especializados. Las observaciones que motivan este análisis provienen precisamente de las preguntas y preocupaciones expresadas por estos mismos profesionales en su labor de campo: ¿Por qué persiste este rechazo a pesar de los esfuerzos educativos? ¿Qué alternativas podrían ser más efectivas? ¿Cómo podemos garantizar una ingesta adecuada de calcio si la leche no es aceptada?

En este contexto, la pregunta de investigación que estructura este ensayo es: ¿por qué las políticas nutricionales en Jujuy continúan priorizando el consumo universal de leche cuando la evidencia científica acumulada durante más de cinco décadas documenta tanto la alta prevalencia de hipolactasia en poblaciones con ancestría indígena como el rechazo global al consumo de leche líquida incluso en poblaciones con alta persistencia de lactasa?

### Magnitud del problema en Jujuy: realidad genética poblacional

La provincia de Jujuy presenta características demográficas y genéticas que la distinguen del resto de Argentina y que son centrales para comprender la inadecuación de políticas nutricionales uniformes. Según el Censo Nacional de 2022, Jujuy tiene la mayor proporción de población que se autoidentifica como indígena en el país: 81.538 personas (10,1% de la población provincial), comparado con un promedio nacional de 2,9%^10^.

Sin embargo, la autoidentificación étnica representa solo una fracción de la ancestría genética indígena real en la población jujeña. Estudios de genética poblacional revelan que la composición genética de Jujuy es sustancialmente más indígena de lo que sugieren las cifras de autoidentificación. Alfaro *et al*.^8^, en su análisis de estructura genética y mezcla en poblaciones urbanas del Noroeste Argentino, documentaron que la población de Jujuy presenta entre 53% y 69% de ancestría genética nativa americana basándose en marcadores autosómicos. Demarchi *et al*.^11^ documentaron una composición genética con predominancia de ancestría nativa americana superior al 60% en las provincias de Jujuy y Salta. Cardoso *et al*.^12^ confirmaron, mediante análisis de ADN mitocondrial, la predominancia de linajes maternos de origen nativo americano en la provincia.

Esta discrepancia entre autoidentificación étnica (10%) y ancestría genética real (53%-69%) refleja patrones de mestizaje asimétrico por género durante el período colonial, producto de la dominación colonial y sus patrones de violencia sexual, con predominancia de madres indígenas y padres europeos, lo que resultó en poblaciones con apellidos europeos y fenotipos mixtos que no se autoidentifican como indígenas, pero que portan variantes genéticas indígenas, incluyendo aquellas relacionadas con la digestión de lactosa.

Si aproximadamente entre el 80% y el 95% de las poblaciones indígenas latinoamericanas estudiadas presentan hipolactasia^6^^,^^13^, y la población de Jujuy tiene entre el 53% y el 69% de ancestría genética indígena, entonces la hipolactasia afecta a la mayoría genética poblacional de Jujuy y no solo al 10% que se autoidentifica como indígena. Esta realidad genética poblacional no se refleja en las políticas nutricionales actuales de la provincia, que adoptan sin modificación las guías nacionales diseñadas para un perfil poblacional diferente.

## Acerca de la investigación

Este ensayo se posiciona en la intersección de tres tradiciones teóricas. Primero, la salud colectiva latinoamericana, que entiende las políticas de salud como fenómenos político-sociales que requieren análisis de las relaciones de poder que las estructuran^14^. Segundo, la medicina evolutiva, que examina cómo las adaptaciones genéticas poblacionales interactúan con ambientes contemporáneos, particularmente, cuando intervenciones de salud pública asumen universalidad biológica inexistente^15^. Tercero, perspectivas decoloniales sobre sistemas alimentarios, que cuestionan la imposición de patrones nutricionales desarrollados en contextos eurooccidentales como normas universales, invisibilizando conocimientos y prácticas alimentarias indígenas^16^.

El análisis integra diversas fuentes documentales. Primero, literatura científica sobre hipolactasia y persistencia de lactasa en poblaciones latinoamericanas e indígenas, integrando tanto estudios clínicos y bioquímicos como estudios genético-poblacionales con datos de ancestría en poblaciones bien caracterizadas. Segundo, análisis de tendencias globales de consumo lácteo utilizando datos de organizaciones internacionales como Food and Agriculture Organization (FAO) y la Organización Mundial de la Salud (OMS), y agencias estadísticas nacionales. Tercero, observaciones etnográficas previas realizadas en Jujuy: el conocimiento aquí presentado emerge de dos proyectos de investigación que, convergentemente, identificaron patrones de rechazo a la leche en contextos distintos de la provincia: uno centrado en salud infantil en instituciones públicas, que incluyó entrevistas con personal de salud en Jujuy (2019-2023) y, otro, que incluyó observaciones etnográficas en comunidades de la Puna jujeña realizadas en el marco de una investigación en curso sobre corporización y nutrición en adolescentes (2020-2024). 

Esta aproximación se constituye en un ensayo analítico que integra diversas fuentes documentales y conocimiento etnográfico acumulado con el propósito de cuestionar la brecha persistente entre conocimiento científico disponible y políticas nutricionales implementadas.

Este ensayo analítico se organiza en torno a cuatro ejes: el primer eje, “*Hipolactasia: formas de consumo y mediaciones culturales en poblaciones con alta ancestría indígena*”, examina la hipolactasia como condición mayoritaria global y particularmente prevalente en poblaciones indígenas latinoamericanas, documentando variabilidad en tolerancia según formas de consumo (productos fermentados, cantidades menores con alimentos) y evidencia genética específica de poblaciones latinoamericanas incluyendo Jujuy. El segundo eje, “*Trayectorias históricas: políticas y recomendaciones de promoción del consumo de leche en América Latina*”**,** analiza cómo, pese a que el problema fue identificado hace más de medio siglo, las políticas continuaron priorizando la distribución de leche mediante estrategias de “adaptación” individual en vez de cuestionar la universalización del modelo lácteo, recuperando políticas nutricionales actuales en Argentina y la brecha entre evidencia disponible y diseño de intervenciones. El tercer eje, “*Prácticas y percepciones alimentarias: observaciones desde las instituciones de salud y educación en Jujuy*”, analiza patrones de rechazo sistemático de leche en programas institucionales y su conceptualización como “resistencia cultural” dentro de los marcos disponibles, contextualizando estos hallazgos con la evidencia genética poblacional y las tendencias globales de consumo lácteo. Por último, el cuarto eje, “*Diseño de políticas públicas nutricionales culturalmente situadas*”, propone recomendaciones específicas para Jujuy basadas en diversificación de fuentes de calcio, reconocimiento de sistemas alimentarios tradicionales, capacitación interdisciplinaria, y consulta comunitaria, alineadas con recomendaciones de organismos internacionales de salud sobre adecuación cultural de guías alimentarias.

## Hipolactasia 

### Formas de consumo y mediaciones culturales en poblaciones con alta ancestría indígena

La capacidad de digerir lactosa más allá de la infancia -conocida como persistencia de lactasa- constituye una adaptación evolutiva minoritaria en la historia humana. La persistencia de lactasa surge de mutaciones genéticas que aparecieron hace aproximadamente 10.000 años en poblaciones con tradiciones pastoriles de larga data, particularmente en el norte de Europa y ciertas regiones de África Oriental^17^^,^^18^. Mientras que todos los mamíferos producen lactasa durante la lactancia para digerir la leche materna, en la mayoría de las poblaciones humanas la producción de esta enzima disminuye después del destete, resultando en hipolactasia en la vida adulta^18^^,^^19^.

A nivel global, entre el 65% y el 70% de la población adulta presenta hipolactasia, constituyendo la condición mayoritaria y esperable desde una perspectiva evolutiva^13^. La distribución geográfica de la persistencia de lactasa -la condición minoritaria- varía dramáticamente: en el norte de Europa la prevalencia de hipolactasia es de entre el 5% y el 15%, mientras que en Asia Oriental alcanza entre el 90% y el 100%^18^. Esta distribución refleja historias evolutivas diferenciadas vinculadas a prácticas pastoriles ancestrales.

Reconceptualizar la hipolactasia como la condición mayoritaria global -en lugar de presentar la persistencia de lactasa como “normal”- tiene implicaciones epistémicas importantes para el diseño de políticas nutricionales. Cuando las guías alimentarias se desarrollan asumiendo persistencia de lactasa como referencia, se naturaliza implícitamente un patrón biológico minoritario derivado de poblaciones del norte de Europa, colocando a la mayoría global en una posición de déficit o desviación que debe “adaptarse” o “corregirse” mediante intervenciones educativas.

### Hipolactasia: mediaciones culturales y formas de consumo

La hipolactasia no es simplemente una preferencia o disgusto cultural, sino una condición biológica con manifestaciones físicas concretas. Individuos con producción reducida de lactasa experimentan síntomas gastrointestinales que se manifiestan típicamente entre 30 minutos y 2 horas después de consumir leche: dolor abdominal, distensión, gases, náuseas y diarrea. Estos síntomas resultan de la fermentación bacteriana de lactosa no digerida en el colon, causando molestias que pueden afectar significativamente el bienestar y las actividades diarias. La severidad varía según la cantidad de lactosa consumida y el grado de deficiencia de lactasa individual^19^. Un vaso típico de leche (200-250 ml) contiene aproximadamente 10-12 gramos de lactosa, cantidad suficiente para provocar síntomas significativos en individuos con hipolactasia, y que es precisamente las porciones que se ofrecen habitualmente en los programas alimentarios escolares.

Sin embargo, la tolerancia a lactosa no depende únicamente de la genética individual o de la cantidad consumida, sino también de cómo se consume. Investigaciones clínicas demuestran que el consumo de lactosa con alimentos sólidos mejora significativamente la tolerancia en personas con hipolactasia debido al vaciado gástrico más lento, que reduce la cantidad de lactosa que llega al intestino delgado por unidad de tiempo^20^. Estudios controlados muestran que mientras 9 de 12 individuos con deficiencia de lactasa experimentaron síntomas al consumir lactosa sola, solo tres presentaron molestias cuando la misma cantidad se consumió con una comida completa^20^. La mayoría de las personas con hipolactasia toleran hasta 12 gramos de lactosa en una sola ingesta cuando se consume con alimentos, cantidad que aumenta a aproximadamente 18 gramos cuando se distribuye durante el día^20^.

Los productos lácteos fermentados representan otra mediación cultural importante. El yogur y el queso contienen cantidades sustancialmente menores de lactosa debido al proceso de fermentación, en el cual las bacterias consumen la lactosa. Además, el yogur proporciona bacterias vivas que producen lactasa, facilitando la digestión^21^^,^^22^. El queso -particularmente variedades maduradas como el cheddar, parmesano o provolone- contiene menos de 1 gramo de lactosa por porción, comparado con los 10-12 gramos de un vaso de leche. Estas características explican por qué estos productos son frecuentemente mejor tolerados incluso por individuos con hipolactasia, y representan adaptaciones culturales desarrolladas históricamente en sociedades que criaban ganado lechero, pero donde la persistencia de lactasa no era universal.

### Evidencia genética en poblaciones latinoamericanas

Como señalan Guimarães Alves *et al*.^13^ en su estudio panamericano, el consumo de lácteos en América se inició con la llegada de los colonizadores europeos, ya que no existe evidencia arqueológica de consumo previo de estos productos en el continente debido a la ausencia de animales domesticados de ordeñe. Este análisis genético encontró que la frecuencia del alelo de persistencia de lactasa se correlaciona directamente con el grado de componente genético europeo, sugiriendo que las poblaciones mestizas americanas presentan prevalencias elevadas de hipolactasia que justificarían una reevaluación de las guías alimentarias aplicadas uniformemente en el continente^13^.

Estudios regionales específicos confirman estos patrones. En Chile, Fernández *et al*.^23^ documentaron que solo el 10% de la población Mapuche presenta persistencia de lactasa, mientras que las poblaciones mestizas chilenas muestran aproximadamente el 40%, porcentaje sustancialmente menor que las poblaciones europeas. En ese mismo estudio, los autores documentaron que el consumo de lácteos en todos los grupos estudiados no alcanzaba los niveles recomendados por las guías nutricionales, con déficits particularmente marcados en las poblaciones indígenas. Los autores encontraron que la asociación entre persistencia de lactasa e ingesta de leche solo se manifestó en dos de las cuatro poblaciones estudiadas, indicando que factores socioeconómicos y preferencias culturales también influyen en los patrones de consumo lácteo más allá de la capacidad digestiva^23^.

En Ecuador, Paz-Y-Miño *et al*.^24^ encontraron una frecuencia de persistencia de lactasa de solo el 12,5% en grupos amerindios con un 82,8% de ancestría nativa americana, observando que las poblaciones pastoriles andinas no desarrollaron variantes genéticas propias para el consumo de leche, a diferencia de las poblaciones pastoriles de Europa y África Oriental que sí experimentaron selección positiva para este rasgo. Estudios en Uruguay^25^ y Brasil^26^ confirman patrones similares de baja persistencia de lactasa correlacionada con ancestría indígena americana.

### Tendencias globales: un fenómeno que trasciende la genética

A la par de estas consideraciones genéticas, las evidencias epidemiológicas revelan un fenómeno global que trasciende diferencias en la persistencia de lactasa: el consumo de leche líquida ha disminuido dramáticamente en las últimas décadas en casi todas las sociedades, incluidas aquellas con las frecuencias más altas de persistencia de lactasa en el mundo.

En EEUU, país con predominancia de ancestría europea del norte, el consumo per cápita de leche ha caído un 47% desde 1975, y actualmente el 90% de la población estadounidense no cumple con las recomendaciones de lácteos del *United States Department of Agriculture* (USDA)^27^. En el Reino Unido, el consumo promedio de leche ha disminuido casi el 50% desde 1974. A nivel de la Unión Europea, se observan tendencias similares de reducción en el consumo de leche líquida en todo el continente^28^. Este patrón se replica en múltiples países desarrollados con las tasas más altas de persistencia de lactasa mundial, sugiriendo que factores que van más allá de la capacidad digestiva -incluyendo cambios en preferencias alimentarias, disponibilidad de alternativas, y transformaciones en estilos de vida- influyen en las decisiones de consumo contemporáneas.

Este rechazo de leche líquida ocurre simultáneamente al aumento dramático en el consumo de productos lácteos fermentados: en EEUU, el consumo de queso aumentó el 179% y el de yogur un 608% desde 1975, precisamente durante el mismo período en que el consumo de leche disminuyó un 47%^29^. Este patrón refuerza la evidencia sobre una mejor tolerancia y aceptación de productos con menor contenido de lactosa.

## Trayectorias históricas

### Políticas y recomendaciones de promoción del consumo de leche en América Latina

El fenómeno político-científico que este eje analiza puede formularse con la siguiente pregunta: ¿cómo es posible que décadas de evidencia no hayan alterado la lógica de los programas lácteos? La respuesta requiere examinar no solo qué decía la evidencia, sino qué hicieron con ella quienes tenían autoridad para traducirla en política. Torún *et al*.^6^, en su exhaustiva revisión de 194 publicaciones sobre malabsorción e intolerancia a la lactosa en América Latina, concluyeron que la correlación entre malabsorción de lactosa e intolerancia clínica a cantidades ordinarias de leche era débil, y que la asunción generalizada de intolerancia a la leche en muchas poblaciones era exagerada. Su recomendación fue continuar los programas de leche con modificaciones menores: porciones reducidas, consumo con alimentos, o uso de productos fermentados. Scrimshaw y Murray^30^, en su revisión dirigida específicamente a audiencias latinoamericanas, argumentaron que para la mayoría de las personas con hipolactasia el consumo moderado de leche no generaba síntomas clínicamente significativos, y que la leche continuaba siendo utilizada exitosamente en programas de alimentación suplementaria infantil en todo el mundo.

Esta postura -que reconocía la prevalencia de hipolactasia pero privilegiaba la continuidad de programas lácteos mediante ajustes individuales- se convirtió en el paradigma dominante que estructuró las políticas nutricionales latinoamericanas durante las siguientes décadas. El marco conceptual asumía que el problema no era la universalización del modelo lácteo en sí, sino la necesidad de educar a las poblaciones sobre cómo consumir leche “apropiadamente”. Este enfoque reflejaba tanto las realidades políticas de la época -la distribución de excedentes lácteos era un componente de la agenda de ayuda alimentaria estadounidense que organismos y sanitaristas locales adoptaron e institucionalizaron en sus propios programas nutricionales^31^^,^^32^- como las limitaciones epistémicas de un momento histórico en el cual las perspectivas decoloniales sobre sistemas alimentarios aún no habían permeado la ciencia nutricional.

### Políticas nutricionales actuales en Argentina: universalización sin diferenciación regional

Las Guías Alimentarias para la Población Argentina (GAPA) recomiendan el consumo diario de 2-3 porciones de lácteos como parte de una alimentación saludable^7^. Estas recomendaciones se implementan de manera uniforme en todo el territorio nacional sin consideraciones específicas para variación genética poblacional regional. Las GAPA reconocen que “los lácteos son fuente de calcio de alta biodisponibilidad y proteínas de alto valor biológico”^7^, posicionando estos productos como componentes esenciales de una dieta adecuada sin mencionar variabilidad poblacional en la capacidad de digerirlos o alternativas equivalentes.

Los programas nutricionales en escuelas y hospitales de Jujuy incluyen con frecuencia la distribución de leche como componente central, alineándose con estas guías nacionales. El Plan Social Nutricional Provincial (PLASONUP) distribuye leche en polvo a comedores escolares para la preparación de desayunos y meriendas^33^. Los servicios de nutrición hospitalaria ofrecen habitualmente té o mate cocido con leche como parte de las bandejas de alimentación para pacientes pediátricos. Estos programas se diseñan e implementan siguiendo protocolos estandarizados que no incorporan consideraciones sobre composición genética poblacional local.

Los marcos normativos que orientan la política nutricional argentina no contemplan la variabilidad genética poblacional como factor relevante^7^, lo que tiende a producir, en la implementación local, un encuadre del rechazo lácteo como déficit educativo o cultural antes que como respuesta fisiológica adaptativa. Esta conceptualización asume que el rechazo refleja desconocimiento o valores culturales inadecuados que deben modificarse, en lugar de considerar que podría constituir una respuesta adaptativa a realidades fisiológicas o una expresión de preferencias alimentarias legítimas arraigadas en tradiciones culinarias regionales. Las capacitaciones al personal de salud enfatizan la importancia de “concientizar” a las familias sobre los beneficios de los lácteos, operando bajo el supuesto de que mayor información conducirá a mayor consumo.

### La brecha persistente entre evidencia científica y diseño de políticas

La evidencia científica acumulada durante más de cinco décadas documenta que: 1) la hipolactasia afecta a la mayoría de la población latinoamericana, con prevalencias del 80% al 95% en poblaciones con alta ancestría indígena^6^^,^^13^; 2) el rechazo a la leche líquida es un fenómeno global que afecta incluso a poblaciones con alta persistencia de lactasa, como evidencian las caídas del 47% al 50% en consumo en EEUU y Reino Unido^27^^,^^28^; 3) los productos lácteos fermentados como el yogur presentan menor contenido de lactosa y son fisiológicamente mejor tolerados por personas con hipolactasia que la leche líquida^21^^,^^22^, aunque la aceptación de estos productos también muestra variabilidad cultural significativa; 4) organismos internacionales reconocen múltiples fuentes de calcio no lácteas igualmente válidas y enfatizan que las guías alimentarias deben desarrollarse en contextos socioculturales específicos^34^^,^^35^; 5) las intervenciones culturalmente adaptadas muestran consistentemente mejores resultados^36^^,^^37^.

Sin embargo, las políticas nutricionales implementadas en Argentina y específicamente en Jujuy no reflejan esta evidencia acumulada. La brecha entre conocimiento disponible y diseño de intervenciones plantea la pregunta: ¿qué factores estructurales impiden que décadas de evidencia científica se traduzcan en políticas más apropiadas? El análisis de esta brecha requiere examinar no solo el contenido científico disponible, sino también los procesos institucionales y epistémicos mediante los cuales el conocimiento se produce, circula, y es -o no es- incorporado en la toma de decisiones de política pública. La oleada de estudios genético-poblacionales de la última década, analizados en el primer eje de este trabajo- no hizo sino confirmar con mayor precisión molecular lo que la investigación clínica de la década de 1960 ya había documentado: a diferencia de poblaciones con ancestría del África subsahariana o del norte de Europa, donde la persistencia de lactasa es más frecuente, en poblaciones con alta ancestría indígena americana la persistencia de lactasa constituye un rasgo minoritario cuya frecuencia decrece proporcionalmente al componente genético indígena, siendo la hipolactasia la condición mayoritaria y fisiológicamente esperable^13^.

### Los silos disciplinarios como obstáculo estructural

Un obstáculo fundamental en la formulación de políticas públicas de salud es la fragmentación del conocimiento científico en compartimentos disciplinarios que operan con relativa independencia. Los genetistas publican sobre persistencia de lactasa en revistas de genética poblacional; los nutricionistas desarrollan guías alimentarias basadas principalmente en bioquímica nutricional; los antropólogos médicos estudian patrones alimentarios culturales y prácticas de consumo en comunidades específicas; los especialistas en salud pública comunitaria analizan adherencia a programas en revistas de su campo. Rara vez profesionales de estas distintas disciplinas son convocados en forma conjunta a mesas de formulación de políticas públicas nutricionales.

El resultado es que existe evidencia científica robusta y relevante para el diseño de intervenciones, pero permanece encapsulada en silos disciplinarios que no se comunican efectivamente entre sí. Las políticas nutricionales terminan reflejando solo una fracción del conocimiento disponible -sobre todo aquel generado dentro de la propia disciplina de la nutrición, con sus marcos conceptuales y metodologías específicas- sin beneficiarse de aportes de genética poblacional, antropología médica, sociología e historia de la alimentación, o epidemiología comparada global, ni de los saberes ancestrales y el conocimiento situado de las propias comunidades. Esta fragmentación no es meramente una deficiencia académica abstracta; tiene consecuencias materiales directas en la efectividad y pertinencia de los programas de salud pública, particularmente en contextos socioculturales diversos como los existentes en la provincia de Jujuy.

La incorporación de evidencia genética poblacional en el diseño de políticas nutricionales requeriría que los comités que desarrollan guías alimentarias incluyeran no solo nutricionistas y médicos, sino también genetistas de poblaciones, epidemiólogos con perspectiva comparada internacional, e historiadores y antropólogos de la alimentación capaces de dar cuenta de cómo las estrategias de adaptación alimentaria se construyen en la intersección de variables biológicas, sociales, económicas y culturales. Pero la diversidad disciplinaria científico-técnica, siendo necesaria, no es suficiente. Como señala Mignolo^16^, la decolonialidad implica romper con la jerarquización del conocimiento y reconocer la multiplicidad de saberes: los saberes ancestrales sobre sistemas alimentarios tradicionales y el conocimiento situado de las propias comunidades merecen integrar la formulación de políticas no como “participación” en mesas diseñadas externamente, sino como diálogo de saberes en pie de igualdad. Los espacios institucionales donde se diseñan políticas nutricionales raramente incorporan esta diversidad -ni disciplinaria ni epistémica- limitando la complejidad del análisis que puede informar las decisiones.

### Raíces históricas: ciencia nutricional y colonialidad del conocimiento

Adicionalmente, merece consideración que la ciencia de la nutrición moderna se desarrolló principalmente en el contexto eurooccidental, específicamente en Alemania, Francia, el Reino Unido y América del Norte durante los siglos XIX y XX, donde ciertos patrones alimentarios, incluyendo el consumo de lácteos, eran predominantes^38^. Este desarrollo histórico ha influido en cómo se conceptualizaron y difundieron globalmente las recomendaciones nutricionales. El consumo de lácteos se asoció frecuentemente con conceptos de modernización y desarrollo nutricional, mientras que los sistemas alimentarios indígenas basados en quinoa, amaranto, legumbres y vegetales nativos recibieron menor atención en las primeras guías nutricionales internacionales o fueron caracterizados como “tradicionales” en un sentido que implicaba necesidad de superación o modernización^16^^,^^32^^,^^38^^,^^39^.

Estos patrones históricos de valoración diferenciada de distintas tradiciones alimentarias reflejan lo que Walter Mignolo caracteriza como colonialidad del conocimiento: la persistencia de jerarquías epistémicas que posicionan formas de conocimiento y prácticas desarrolladas en Europa y América del Norte como universales y científicas, mientras que conocimientos y prácticas de origen no-europeo se consideran locales, tradicionales, o culturales, categorías que implican menor validez o aplicabilidad general^16^. En el campo de la nutrición, esta tensión epistémica se manifiesta en la tendencia a naturalizar patrones alimentarios europeos como base para recomendaciones “universales”, invisibilizando tanto la variación genética poblacional que afecta la digestibilidad de ciertos alimentos como la sofisticación nutricional de sistemas alimentarios que desarrollaron estrategias alternativas para alcanzar adecuación nutricional sin lácteos.

La anulación de estas características poblacionales y conocimientos alimentarios locales en el diseño de políticas nutricionales uniformes reproduce lo que Mignolo^16^ identifica como un efecto central del colonialismo: la subalternización de sistemas de conocimiento y formas de vida de poblaciones colonizadas, que perdura mucho después del fin del colonialismo político formal. Reconocer estas dinámicas históricas no implica descartar la ciencia nutricional contemporánea, que ha generado conocimientos valiosos sobre requerimientos nutricionales, biodisponibilidad de nutrientes, y relaciones entre dieta y salud, sino contextualizarla como producto de procesos históricos específicos cuyos sesgos merecen análisis críticos cuando se aplican en contextos poblacionales diferentes de aquellos donde se originaron.

### Ausencia de participación comunitaria en el diseño de políticas

El análisis de silos disciplinarios se ha centrado hasta aquí en la falta de comunicación entre diferentes campos académicos y técnicos. Sin embargo, existe una ausencia aún más fundamental: la ciudadanía, entendida no solo como destinataria de políticas, sino como sujeto activo que demanda, interpela y produce saberes sociales en torno a la alimentación, el cuerpo y la salud^40^^,^^41^. Desde la perspectiva de la salud colectiva^14^, esta omisión invisibiliza el papel que históricamente han tenido foros comunitarios, organizaciones sociales, asociaciones indígenas, agrupaciones de madres y familias, y espacios de participación local en la construcción de diagnósticos sobre la inadecuación de ciertas intervenciones nutricionales^39^^,^^40^.

Estos espacios no solo “opinan” sobre políticas diseñadas por expertos, sino que producen conocimiento situado^42^^,^^43^, transversal a las disciplinas académicas, que muchas veces antecede y tensiona a la evidencia científica formal. Las madres de comunidades de la Puna que reportan que la leche “les cae mal” a sus hijos no están expresando ignorancia nutricional que debe corregirse mediante educación, sino conocimientos empíricos basados en observación sistemática de síntomas gastrointestinales que la ciencia médica posteriormente caracterizó como manifestaciones de hipolactasia. Los docentes que observan el rechazo persistente al mate cocido con leche en comedores escolares no están reportando una anomalía cultural, sino documentando un patrón epidemiológico que refleja realidades fisiológicas poblacionales.

La incorporación efectiva de estas voces en el diseño de políticas nutricionales requeriría mecanismos institucionales de diálogo y co-diseño con organizaciones locales, y valoración del conocimiento experiencial al mismo nivel que la evidencia científica formal^39^^,^^40^. Requeriría reconocer que las comunidades que rechazan sistemáticamente ciertos alimentos ofrecidos en programas institucionales merecen programas diseñados en diálogo con sus realidades fisiológicas, preferencias culturales, y conocimientos sobre sus propios cuerpos y sistemas alimentarios^39^^,^^40^.

## Prácticas y percepciones alimentarias

### Observaciones desde las instituciones de salud y educación en Jujuy

En instituciones de salud pública que atienden población de toda la provincia, el personal describe cómo los niños internados frecuentemente no consumen el mate cocido o té con leche que se les ofrece en el desayuno o merienda, a pesar de los consejos nutricionales proporcionados por profesionales sobre la importancia de la leche para la salud infantil. En escuelas que ofrecen desayunos y meriendas escolares a través del Plan Social Nutricional Provincial (PLASONUP), el patrón se repite de manera consistente: el té o mate cocido con leche -bebida tradicional en los programas alimentarios escolares- es frecuentemente rechazado por los niños.

Los marcos interpretativos institucionales vigentes tienden a conceptualizar este rechazo como “resistencia cultural” asociada a escasa información nutricional en las familias, orientando las intervenciones hacia la promoción del consumo de lácteos como fuente prioritaria de calcio. Esta conceptualización es comprensible dado el estado del conocimiento disponible en décadas anteriores, pero no incorpora la evidencia acumulada sobre variación genética poblacional en la digestibilidad de la lactosa ni sobre fuentes alternativas de calcio igualmente válidas. Desde esta perspectiva, el rechazo se interpreta como un obstáculo que puede resolverse mediante información adicional, antes que como una señal que podría indicar la necesidad de actualizar y diversificar el contenido de las propias intervenciones nutricionales.

Sin embargo, cuando estas observaciones se contextualizan en el marco de la evidencia genética poblacional presentada en el primer eje, que documenta que del 53% al 69% de la población jujeña presenta ancestría indígena^8^^,^^11^ y que del 80% al 95% de poblaciones indígenas latinoamericanas presentan hipolactasia^13^^,^^6^, el patrón de rechazo adquiere una interpretación alternativa. Lo que se registra institucionalmente como “resistencia cultural” podría constituir, al menos parcialmente, una respuesta fisiológica a molestias digestivas experimentadas después del consumo de cantidades estándar de leche líquida. Los síntomas característicos de hipolactasia -dolor abdominal, distensión, náuseas- se manifiestan entre 30 minutos y 2 horas después del consumo^20^, precisamente el período en que las niñas y los niños están en contextos institucionales donde el personal puede observar el malestar, pero no necesariamente asociarlo causalmente con el consumo de leche ocurrido previamente.

Esta desconexión interpretativa -donde síntomas fisiológicos se registran como preferencias culturales irracionales- refleja las consecuencias prácticas de los silos disciplinarios y las jerarquías epistémicas analizados en el segundo eje. Los marcos nutricionales vigentes en la formación de salud pública no incorporan evidencia de genética poblacional, ni perspectivas de antropología médica, sociología o historia de la alimentación, ni el conocimiento situado de las propias comunidades, lo que resulta en una ausencia de herramientas conceptuales para interpretar el rechazo sistemático como posible manifestación tanto de variación genética regional como de saberes legítimos sobre los propios cuerpos y sistemas alimentarios. La ausencia de formación interdisciplinaria que integre estas dimensiones resulta en que patrones epidemiológicos y culturales significativos permanezcan invisibilizados.

### El rechazo como conocimiento situado: hacia una educación nutricional basada en evidencia interdisciplinaria

La convergencia entre las observaciones documentadas en instituciones de salud pública y hallazgos etnográficos independientes en comunidades de la provincia sugiere que el rechazo a la leche constituye un patrón regional amplio que merece interpretación más allá de marcos que lo caracterizan como ignorancia nutricional o irracionalidad cultural. Las madres que reportan que la leche “les cae mal” a sus hijos no están expresando creencias infundadas, sino conocimiento empírico basado en observación sistemática de síntomas que la ciencia médica posteriormente caracterizó como manifestaciones de hipolactasia^19^^,^^20^. Los niños y las niñas que rechazan el mate cocido con leche en comedores escolares no están siendo “difíciles” o resistiendo por capricho, sino respondiendo a molestias digestivas reales que experimentan después del consumo.

Reconceptualizar el rechazo a la leche como expresión de conocimiento situado^42^^,^^43^, más que como déficit educativo que requiere corrección, tiene implicaciones importantes para el diseño de políticas nutricionales y programas educativos. La educación nutricional y la salud escolar seguirían siendo fundamentales, pero su contenido se reorientaría: en lugar de promover el consumo de lácteos como vía única o prioritaria para alcanzar requerimientos de calcio, se educaría sobre múltiples fuentes nutricionalmente equivalentes que respeten las realidades fisiológicas documentadas^35^^,^^37^. Este enfoque reconoce las señales que las comunidades están enviando sobre qué alimentos funcionan para sus cuerpos, integrando ese conocimiento situado en programas educativos basados en evidencia genética poblacional. 

Los profesionales de educación para la salud, nutricionistas escolares, médicos, y equipos de salud pública son aliados esenciales en esta transformación. Su experticia en comunicación efectiva, diseño de intervenciones educativas y conocimiento de las realidades locales resulta invaluable para traducir evidencias genéticas poblacionales en programas culturalmente apropiados y nutricionalmente equivalentes. El objetivo no es cuestionar la importancia de la educación nutricional, sino actualizarla con evidencia interdisciplinaria que permita diseñar intervenciones más efectivas que trabajen con las realidades fisiológicas y culturales de las poblaciones. Este reposicionamiento implica un cambio: pasar de ver a las comunidades como poblaciones que necesitan ser educadas y adoptar comportamientos predefinidos, a reconocerlas como sujetos con conocimiento válido sobre sus propias necesidades nutricionales que deben integrar el diseño colaborativo de políticas públicas^37^^,^^40^.

La evidencia genética poblacional, las tendencias globales de consumo, y las observaciones convergentes sugieren que el problema no radica en las comunidades que rechazan la leche, sino en políticas nutricionales que universalizan un modelo alimentario desarrollado para poblaciones con características genéticas diferentes. La pregunta apropiada no es “¿cómo hacer que las comunidades jujeñas consuman más leche?”, sino “¿cómo diseñar políticas nutricionales y programas educativos que respeten las realidades fisiológicas y culturales de la población mayoritaria de Jujuy, que garanticen adecuación de calcio y otros nutrientes esenciales?”

## Diseño de políticas públicas nutricionales culturalmente situadas

### Fuentes alternativas de calcio: reconocimiento oficial internacional

La FAO y la OMS enfatizan que las guías alimentarias deben desarrollarse en contextos socioculturales específicos^34^, y reconocen formalmente que el calcio puede obtenerse adecuadamente de múltiples fuentes no lácteas^35^. Este reconocimiento internacional proporciona fundamento para políticas nutricionales que diversifiquen estrategias para alcanzar requerimientos de calcio más allá de la dependencia exclusiva de productos lácteos.

Una alimentación variada puede proveer calcio adecuado sin depender exclusivamente de lácteos. Las legumbres -incluyendo garbanzos y porotos blancos- representan fuentes accesibles y culturalmente familiares de calcio en la región. Los vegetales de hoja verde disponibles localmente en la región andina -hojas de quinua, hojas de nabo, berza y acelga- aportan cantidades significativas de calcio. Las dietas tradicionales andinas han proporcionado históricamente calcio a través de granos como la quinua (47-80 mg por 100g), el amaranto o kiwicha (150-200 mg), el tarwi o chocho (50-80 mg), y tubérculos deshidratados^44^^,^^45^.

Las poblaciones altoandinas desarrollaron prácticas tradicionales de fortificación alimentaria como agregar cal, lejía o cenizas -compuestos alcalinos que incluyen hidróxido de calcio- durante la cocción de quinua y otros granos, incrementando sustancialmente el contenido disponible de calcio y mejorando su biodisponibilidad^44^^,^^45^. La deshidratación de papa mediante ciclos naturales de congelación nocturna y exposición solar en la Puna -proceso que da origen al chuño- constituye otro ejemplo de tecnología alimentaria andina que mejora la conservación y el perfil nutricional de alimentos tradicionales^46^. Estas prácticas evidencian conocimiento empírico sobre equilibrio mineral dietético y procesamiento alimentario, demostrando que las poblaciones andinas desarrollaron mecanismos propios de enriquecimiento y conservación alimentaria antes de la introducción de productos industriales^39^.

Reorientar políticas hacia el fortalecimiento de alimentos locales ricos en calcio favorecería mejor la adecuación fisiológica y cultural, contribuyendo simultáneamente a la soberanía alimentaria y al reconocimiento de saberes nutricionales tradicionales. Como señalan estudios sobre intervenciones culturalmente adaptadas, las modificaciones graduales que respetan los sistemas de creencias y estilos de vida existentes resultan más efectivas y sostenibles que los cambios radicales impuestos externamente^36^^,^^37^.

### Hacia políticas nutricionales basadas en prevalencia poblacional

La fortificación obligatoria de sal con yodo establecida en 1967^47^^,^^48^ y de harinas con ácido fólico implementada desde 2002^49^ no requirieron testeo individual previo, sino que se implementaron sobre la base de prevalencias conocidas de deficiencias en la población general. Estas intervenciones demostraron efectividad significativa: la fortificación con ácido fólico se asoció con reducción del 56% en mortalidad por anencefalia y del 67% en mortalidad por espina bífida en los años posteriores a su implementación^50^.

Desde la salud colectiva, la distinción entre estrategias de alto riesgo que requieren identificación individual y estrategias poblacionales basadas en prevalencias epidemiológicas^51^ puede ser recuperada críticamente para argumentar que, cuando una condición afecta a una proporción sustancial de la población -como es el caso de la hipolactasia en poblaciones con ancestría indígena, donde las prevalencias alcanzan del 80% al 95%^8^^,^^13^- las adaptaciones de políticas alimentarias pueden implementarse sin necesidad de detección individual, garantizando acceso universal a alternativas nutricionales adecuadas sin imponer la carga del diagnóstico médico. Esta recuperación no implica adoptar acríticamente el paradigma de la salud pública, sino utilizar sus propias herramientas para cuestionar intervenciones que, paradójicamente, individualizan un problema de base poblacional.

### Diversificación de fuentes de calcio en programas institucionales

Los programas alimentarios escolares y hospitalarios podrían reformularse para ofrecer múltiples opciones para alcanzar los requerimientos de calcio en vez de depender centralmente de leche líquida como bebida. Esta diversificación no representa un costo adicional significativo, sino una redistribución de recursos ya asignados hacia opciones con mayor probabilidad de consumo efectivo y mejor tolerancia fisiológica^35^.

Los productos lácteos fermentados como quesos maduros (cheddar, parmesano, provolone) y yogur con cultivos vivos contienen cantidades sustancialmente menores de lactosa debido al proceso de fermentación y son generalmente mejor tolerados digestivamente. El queso maduro contiene menos de 1 gramo de lactosa por porción comparado con los 10-12 gramos de un vaso de leche líquida^22^. Sin embargo, la aceptación de yogur puede variar según contexto poblacional, condiciones climáticas y marcos culturales locales. Los programas deberían evaluar la aceptación de diferentes productos lácteos fermentados según características específicas de cada población atendida.

La promoción de alimentos andinos ricos en calcio como quinua, amaranto, y verduras de hoja verde representa una estrategia culturalmente apropiada y nutricionalmente adecuada. El fortalecimiento de prácticas tradicionales como el uso de cal durante la cocción de granos -que incrementa el contenido disponible de calcio- constituye un ejemplo de cómo las políticas nutricionales pueden trabajar con conocimientos tradicionales en lugar de sustituirlos por modelos externos^44^^,^^45^. Esta aproximación reconoce la sofisticación de tecnologías alimentarias andinas desarrolladas a lo largo de milenios para optimizar la nutrición en ambientes de altura^39^.

Las preparaciones que incorporan cantidades menores de leche cocida con otros ingredientes, como arroz con leche, flanes, o sopas enriquecidas, representan una opción intermedia. La evidencia clínica indica mejor tolerancia de lactosa cuando se consume en cantidades menores y combinada con alimentos sólidos que ralentizan el vaciado gástrico^20^, sugiriendo que estas preparaciones podrían tener mejor aceptación. Estas preparaciones podrían priorizarse en programas piloto que evalúen sistemáticamente aceptación, tolerancia digestiva y adherencia en distintos contextos antes de implementación provincial. La ventaja de este enfoque es triple: aprovecha la mejor tolerancia de lactosa en preparaciones cocidas con otros alimentos; permite mantener cierto aporte lácteo para quienes lo toleran sin imponer consumo de leche líquida a quienes presentan síntomas; y facilita la transición dentro de los programas institucionales existentes sin requerir un rediseño completo de menús ni una inversión adicional significativa de recursos.

### Capacitación profesional interdisciplinaria como estrategia central

La brecha entre el conocimiento disponible -tanto científico como situado en las comunidades- y las políticas implementadas no se resolverá únicamente mediante modificaciones de menús escolares o diversificación de opciones alimentarias. Requiere transformación de las capacidades institucionales para integrar evidencia de múltiples disciplinas y para establecer procesos genuinos de diálogo con las poblaciones destinatarias. Los programas de formación continua para nutricionistas, médicos y personal de salud pública en Jujuy podrían incorporar módulos sobre composición genética específica de la población jujeña, variación genética poblacional en la digestión de lactosa, tendencias globales en consumo de lácteos, enfoques de nutrición culturalmente situados, lectura crítica de literatura científica fuera de la disciplina de origen, y habilidades para la escucha activa y el diálogo intercultural con las comunidades destinatarias de las intervenciones.

Crucialmente, estas capacitaciones deberían fortalecer el trabajo interdisciplinario, rompiendo los silos que limitan la traducción del conocimiento disponible en mejores políticas públicas. La capacitación no debe concebirse como transmisión unidireccional de información técnica, sino como desarrollo de capacidades para integrar conocimientos de genética poblacional, antropología médica, epidemiología comparada y nutrición clínica con saberes ancestrales y el conocimiento situado de las comunidades, en marcos analíticos coherentes que informen decisiones políticas. Esto requiere no solo adquisición de contenidos específicos, sino desarrollo de habilidades para el diálogo interdisciplinario e intercultural efectivo.

### Consulta comunitaria y co-diseño de programas como imperativo ético y pragmático

Las intervenciones nutricionales se benefician sustancialmente cuando se desarrollan en colaboración con las comunidades destinatarias en lugar de diseñarse externamente e implementarse verticalmente. Este principio no es meramente un ideal participativo abstracto, sino una estrategia pragmática basada en evidencia sobre efectividad de intervenciones.

Sesiones de consulta con organizaciones comunitarias indígenas y mestizas sobre preferencias alimentarias pueden identificar qué alimentos son culturalmente aceptables, accesibles localmente, y nutricionalmente adecuados. La identificación participativa de alimentos puede revelar opciones que el personal técnico externo podría no considerar, incorporando conocimiento tradicional sobre alimentos nutritivos locales que ha sostenido la salud poblacional durante generaciones. El co-diseño de programas piloto permite evaluar aceptación y adherencia en condiciones reales antes de implementación a mayor escala, reduciendo el riesgo de inversión significativa de recursos en intervenciones que posteriormente enfrentan rechazo comunitario^41^.

Este enfoque no solo mejora la probabilidad de éxito programático, sino que también aborda el respeto a la autonomía y el conocimiento de las comunidades. Reconoce que las poblaciones destinatarias no son objetos pasivos de intervenciones diseñadas por expertos, sino sujetos con conocimiento válido sobre sus propias necesidades, preferencias, y realidades que deben incorporarse activamente en el diseño de políticas que las afectan.

### Actualización de guías alimentarias provinciales: reconociendo especificidad regional

Las GAPA operan a nivel nacional con recomendaciones uniformes que no consideran variación genética poblacional regional. Jujuy podría desarrollar un suplemento provincial a las GAPA nacionales que reconozca la composición genética específica de su población, las tradiciones alimentarias regionales, la equivalencia nutricional de diversas fuentes de calcio, y la flexibilidad en alcanzar requerimientos nutricionales mediante múltiples vías. Esta actualización no requeriría contradecir las guías nacionales, sino complementarlas con especificaciones regionales que reflejen realidades locales.

El suplemento provincial podría explicitar que mientras las GAPA nacionales recomiendan 2-3 porciones diarias de lácteos, en Jujuy estos requerimientos de calcio pueden alcanzarse mediante combinaciones de productos lácteos fermentados (quesos, yogur según tolerancia y preferencia local), alimentos andinos tradicionales ricos en calcio (quinua, amaranto, vegetales de hoja verde), legumbres, y preparaciones que incorporan cantidades menores de leche cocida con otros alimentos. Esta especificación respetaría el marco nacional mientras proporcionaría orientación práctica apropiada para la realidad genética y cultural jujeña.

La actualización serviría como modelo para otras provincias argentinas con alta ancestría indígena, como Salta, Formosa y Chaco, demostrando que las guías alimentarias nacionales pueden adaptarse regionalmente sin fragmentar el sistema de salud pública. Este enfoque reconoce que Argentina es un país con considerable diversidad genética y cultural regional que merece consideración en el diseño de políticas nutricionales, en lugar de asumir que recomendaciones diseñadas para el perfil poblacional de Buenos Aires o la región pampeana aplican uniformemente en el Noroeste andino.

### Monitoreo y evaluación con indicadores centrados en resultados nutricionales

Los programas nutricionales deben evaluarse según su efectividad real en mejorar el estado nutricional, no según adherencia a modelos específicos de intervención. El indicador apropiado no es “consumo de leche” sino “adecuación de calcio” y “salud ósea”. Este cambio de enfoque requiere reformulación de los sistemas de monitoreo y evaluación programática.

Los indicadores apropiados incluirían niveles de calcio sérico y marcadores de salud ósea en muestras poblacionales, ingesta total de calcio de todas las fuentes evaluadas mediante recordatorios dietéticos de 24 horas o encuestas de frecuencia de consumo, adherencia y aceptación de los programas alimentarios, medible por medio de tasas de consumo efectivo de alimentos ofrecidos, y satisfacción comunitaria con las opciones disponibles evaluada mediante encuestas a familias y estudiantes. Este conjunto de indicadores proporcionaría información sobre si los programas están logrando sus objetivos nutricionales reales, independientemente de si lo hacen mediante leche, quesos, quinua, o combinaciones de fuentes.

El enfoque en resultados nutricionales en lugar de adherencia a alimentos específicos permite flexibilidad programática basada en evidencia de efectividad. Si los datos muestran que los programas que ofrecen diversidad de fuentes de calcio logran mejor adecuación nutricional y mayor adherencia que programas centrados en leche líquida -como la evidencia presentada en este ensayo sugiere que sería el caso- entonces las políticas pueden ajustarse basándose en resultados observados. El monitoreo centrado en resultados nutricionales también permite identificar qué combinaciones de alimentos funcionan mejor en diferentes contextos (urbano vs rural, diferentes altitudes, diferentes composiciones étnicas de la población escolar), informando refinamiento continuo de intervenciones.

### Investigación local colaborativa interdisciplinaria como fundamento para políticas basadas en evidencia

Finalmente, el sistema de salud de Jujuy, en colaboración con universidades locales y equipos interdisciplinarios que incluyan profesionales de nutrición, genética, antropología médica y salud pública comunitaria, podría desarrollar investigación que documente la prevalencia real de persistencia de lactasa en diferentes poblaciones de la provincia, los patrones de consumo lácteo actual desagregados por región y autoidentificación étnica, la evaluación del estado nutricional de calcio y salud ósea en niños y adolescentes, y la efectividad comparativa de diferentes intervenciones nutricionales implementadas como programas piloto.

Estos datos locales generados colaborativamente entre disciplinas fortalecerían aún más la base de evidencia para políticas regionalmente apropiadas y demostrarían el valor del trabajo interdisciplinario en la generación de conocimiento aplicable a decisiones de política pública. La investigación local también construiría capacidad institucional provincial para evaluación continua de programas y refinamiento basado en evidencia, reduciendo la dependencia de modelos diseñados externamente que pueden no ajustarse a realidades locales.

La generación de evidencia local sobre Jujuy específicamente -en lugar de extrapolar estudios de otras poblaciones- proporcionaría fundamento robusto para justificar adaptaciones regionales de políticas nacionales. Permitiría responder con datos propios cuando se cuestione la pertinencia de modificar guías nacionales, demostrando mediante evidencia empírica local que las características genéticas y culturales de la población jujeña justifican enfoques diferenciados. Esta investigación también podría identificar variación intraprovincial, es decir, diferencias entre poblaciones de la Puna, valles, y zona urbana, que demandaría el diseño de intervenciones aún más específicamente adaptadas a realidades micro-regionales.

Es necesario reconocer, sin embargo, que la implementación de estas propuestas enfrenta obstáculos estructurales que van más allá de la falta de enunciación. Quienes estudian críticamente las políticas públicas de salud en América Latina vienen señalando desde hace décadas las dificultades de integrar diversas formas de conocimiento y subjetividades en la práctica institucional^14^^,^^39^^,^^40^: las relaciones de poder que estructuran los espacios de toma de decisiones, la resistencia de los sistemas institucionales al cambio, las tensiones entre lógicas técnico-burocráticas y lógicas comunitarias, y la complejidad de sostener procesos genuinos de diálogo intercultural en contextos de desigualdad estructural. Reconocer estas dificultades no invalida las propuestas, sino que demanda estrategias de implementación que las anticipen y que construyan, gradualmente y con las comunidades, las condiciones institucionales y políticas necesarias para que el cambio sea posible y sostenible

## Conclusiones

El análisis presentado en este ensayo ha demostrado que la desconexión entre políticas nutricionales universales y la realidad genética y los saberes de las poblaciones con alta ancestría indígena en Jujuy no es resultado de falta de conocimiento científico. Los cuatro ejes analíticos desarrollados convergen en evidenciar que medio siglo después de que la investigación científica documentara extensamente la prevalencia de hipolactasia en América Latina, las políticas nutricionales argentinas continúan naturalizando el patrón metabólico minoritario europeo como norma universal.

La evidencia presentada en el primer eje sobre la hipolactasia como condición mayoritaria mundial (del 65% al 70% de la población global), junto con los datos genéticos que muestran del 53% al 69% de ancestría indígena en Jujuy, establece que las políticas alimentarias en esta provincia enfrentan un desafío de salud pública poblacional, no una cuestión de atención a minorías. Argentina ha implementado exitosamente políticas nutricionales basadas en prevalencias poblacionales sin requerir testeo individual, como la fortificación obligatoria de sal con yodo y de harinas con ácido fólico responden a deficiencias conocidas en la población general sin exigir diagnóstico médico previo. El mismo principio puede aplicarse al diseño de programas alimentarios que reconozcan la alta prevalencia probable de hipolactasia en Jujuy, ofreciendo diversidad de fuentes de calcio sin imponer la carga de diagnóstico individual para acceder a opciones fisiológicamente apropiadas.

El segundo eje documentó cómo la literatura científica desde la década de 1960 reconoció la hipolactasia en poblaciones latinoamericanas, pero recomendó la continuidad de programas lácteos mediante “adaptaciones individuales”. Cincuenta años después, esta aproximación ha demostrado sus limitaciones: las instituciones de salud y educación en Jujuy continúan interpretando el rechazo sistemático de leche líquida como “resistencia cultural” que requiere educación nutricional, invisibilizando la posibilidad de respuesta fisiológica a malestar digestivo. Como reveló el tercer eje, el conocimiento situado de madres y familias que reportan que la leche “les cae mal” a sus hijos constituye observación empírica válida sobre síntomas de hipolactasia, no ignorancia que requiera corrección educativa. Recentrar esta observación comunitaria como conocimiento válido en lugar de déficit cognitivo representa un cambio epistemológico fundamental hacia el respeto de saberes situados.

La transformación de políticas nutricionales en Jujuy requiere romper los silos disciplinarios que han impedido que evidencia científica disponible desde hace décadas informe el diseño de intervenciones. Como propone el cuarto eje, esto demanda capacitación interdisciplinaria del personal de salud pública que desarrolle capacidades para integrar conocimientos de genética poblacional, antropología médica, epidemiología comparada y nutrición clínica. Crucialmente, requiere procesos genuinos de consulta y co-diseño con las comunidades destinatarias, reconociendo que las poblaciones no son objetos pasivos de intervenciones diseñadas externamente, sino sujetos con conocimientos válidos sobre sus propias necesidades, preferencias y realidades que debe incorporarse activamente en el diseño de políticas que las afectan.

El reconocimiento de sistemas alimentarios andinos, que durante milenios han proporcionado nutrición adecuada mediante quinua, amaranto, hojas de quinua, tubérculos, y prácticas de fortificación con compuestos alcalinos, no es romanticización del pasado sino valoración informada de tecnologías alimentarias sofisticadas desarrolladas en condiciones ambientales específicas. Las políticas nutricionales culturalmente situadas no representan un abandono de objetivos de salud pública, sino una estrategia más efectiva para alcanzarlos. La meta no es consumo de leche sino adecuación de calcio y salud ósea. Si esta meta se logra mejor mediante diversidad de fuentes que mediante dependencia exclusiva de un alimento que una proporción sustancial de la población no tolera digestivamente ni prefiere culturalmente, entonces las políticas basadas en evidencia deben priorizar los resultados nutricionales sobre la adherencia a modelos alimentarios importados.

Jujuy tiene la oportunidad de desarrollar un modelo de políticas nutricionales que reconozca explícitamente la composición genética de su población, que valore los sistemas alimentarios tradicionales andinos como fuentes legítimas de nutrición adecuada, que implemente programas basados en prevalencias poblacionales siguiendo el modelo exitoso de otras intervenciones de salud pública argentinas, y que construya genuina colaboración entre profesionales de salud pública y comunidades destinatarias. Este modelo no solo mejoraría los resultados nutricionales en la provincia, sino que podría servir de referencia para otras regiones argentinas con alta ancestría indígena, demostrando que las guías alimentarias nacionales pueden adaptarse regionalmente para reflejar la diversidad genética y cultural del país sin fragmentar el sistema de salud pública. Lo que está en juego no es simplemente si los niños y las niñas de Jujuy toman leche en el desayuno escolar, sino si el sistema de salud pública argentino es capaz de integrar evidencia científica disponible, respetar el conocimiento y las preferencias comunitarias, y diseñar políticas nutricionalmente equivalentes que sirvan efectivamente a todas las poblaciones del país.

## References

[1] Alzate H, Ramírez E, Echeverry MT (1968). Intolerancia a la lactosa en un grupo de estudiantes de medicina. Antioquia Médica.

[2] Alzate H, González H, Guzmán J (1969). Lactose intolerance in South American Indians. American Journal of Clinical Nutrition.

[3] Vasconcellos D, Gonçalves A (1968). Deficiência de lactase en adultos. Arquivos de Gastroenterologia.

[4] Bayless TM, Rosensweig NS (1966). A racial difference in incidence of lactase deficiency: A survey of milk intolerance and lactase deficiency in healthy adult males. JAMA.

[5] Paige DM, Bayless TM, Ferry GD, Graham GG (1971). Lactose malabsorption and milk rejection in Negro children. Johns Hopkins Medical Journal.

[6] Torún B, Solomons NW, Viteri FE (1979). Lactose malabsorption and lactose intolerance: implications for general milk consumption in Latin America. Archivos Latinoamericanos de Nutrición.

[7] Argentina, Ministerio de Salud (2016). Guías Alimentarias para la Población Argentina.

[8] Alfaro EL, Dipierri JE, Gutierrez NI, Vullo CM (2005). Genetic structure and admixture in urban populations of the Argentine North-West. Annals of Human Biology.

[9] Toca MC, Fernández A, Orsi M, Tabacco O, Vinderola G (2022). Intolerancia a la lactosa: mitos y verdades, Actualización. Archivos Argentinos de Pediatría.

[10] Instituto Nacional de Estadística y Censos (INDEC) (2024). Censo Nacional de Población, Hogares y Viviendas 2022: Resultados definitivos: pueblos indígenas u originarios.

[11] Demarchi DA, Claria DM, Dipierri JE, Gardenal CN (2000). Genetic structure of native Andean populations from Argentina inhabiting different altitudes. Human Biology.

[12] Cardoso S, Palencia-Madrid L, Valverde L, Alfonso-Sanchez MA, Gomez-Perez L, Alfaro E (2013). Mitochondrial DNA control region data reveal high prevalence of Native American lineages in Jujuy province, NW Argentina. Forensic Science International: Genetics.

[13] Guimarães Alves AC, Sukow NM, Adelman Cipolla G, Mendes M, Leal TP, Petzl-Erler ML (2021). Tracing the distribution of European lactase persistence genotypes along the Americas. Frontiers in Genetics.

[14] Paim JS (2021). Desafíos para la salud colectiva en el siglo XXI.

[15] Williams GC, Nesse RM (1991). The dawn of Darwinian medicine. Quarterly Review of Biology.

[16] Mignolo WD, Lander E (2000). La colonialidad del saber: eurocentrismo y ciencias sociales, Perspectivas latinoamericanas.

[17] Gerbault P, Roffet-Salque M, Evershed RP, Thomas MG (2013). How long have adult humans been consuming milk?. IUBMB Life.

[18] Ségurel L, Bon C (2017). On the evolution of lactase persistence in humans. Annual Review of Genomics and Human Genetics.

[19] Mattar R, de Campos Mazo DF, Carrilho FJ (2012). Lactose intolerance: diagnosis, genetic, and clinical factors. Clinical & Experimental Gastroenterology.

[20] Martini MC, Savaiano DA (1988). Reduced intolerance symptoms from lactose consumed during a meal. American Journal of Clinical Nutrition.

[21] Savaiano DA, AbouElAnouar A, Smith DE, Levitt MD (1984). Lactose malabsorption from yogurt, pasteurized yogurt, sweet acidophilus milk, and cultured milk in lactase-deficient individuals. American Journal of Clinical Nutrition.

[22] Hertzler SR, Clancy SM (2003). Kefir improves lactose digestion and tolerance in adults with lactose maldigestion. Journal of the American Dietetic Association.

[23] Fernández CI, Montalva N, Arias M, Hevia M, Moraga ML, Flores SV (2016). Lactase non-persistence and general patterns of dairy intake in indigenous and mestizo Chilean populations. American Journal of Human Biology.

[24] Paz-Y-Miño C, Burgos G, López-Cortés A, Herrera C, Gaviria A, Tejera E (2016). A study of the molecular variants associated with lactase persistence in different Ecuadorian ethnic groups. American Journal of Human Biology.

[25] Gaudin RGN, Figueiro G, Flores-Gutiérrez S, Mut P, Vega-Requena Y, Luna-Andrada L (2023). DNA polymorphisms associated with lactase persistence, self-perceived symptoms of lactose intolerance, milk and dairy consumption, and ancestry, in the Uruguayan population. American Journal of Human Biology.

[26] Friedrich DC, Santos SE, Ribeiro-dos-Santos ÂK, Hutz MH (2012). Several different lactase persistence associated alleles and high diversity of the lactase gene in the admixed Brazilian population. PLoS One.

[27] United States Department of Agriculture, Economic Research Service (2022). Fluid Milk Consumption Continues Downward Trend, Proving Difficult to Reverse.

[28] Behera S (2024). How is milk utilisation trending in the UK?.

[29] United States Department of Agriculture, Economic Research Service (2025). Dairy Data.

[30] Scrimshaw NS, Murray EB (1988). The acceptability of milk and milk products in populations with a high prevalence of lactose intolerance. American Journal of Clinical Nutrition.

[31] Brinkmann S, Buschini J (2021). Presentación. La “cuestión de la leche” en América Latina: expertos, mercados y políticas públicas en el siglo XX. História, Ciência & Saúde-Manguinhos.

[32] Reyna C (2023). “Mejor nutrición no significa necesariamente más comida”: educación alimentaria y fomento agrícola en Argentina (1960-1970). Revista Ciencias de la Salud.

[33] Argentina, Gobierno de Jujuy (2003). Ley 5343: Plan Social Nutricional Provincial (PLASONUP).

[34] Food and Agriculture Organization, World Health Organization (1998). Preparation and use of food-based dietary guidelines: Report of a joint FAO/WHO consultation.

[35] Food and Agriculture Organization, World Health Organization (2001). Human vitamin and mineral requirements: Report of a joint FAO/WHO expert consultation, Bangkok, Thailand.

[36] Winham DM (2009). Culturally Tailored Foods and Cardiovascular Disease Prevention. American Journal of Lifestyle Medicine.

[37] Vincze L, Wilson A, Burgess C, Strugnell C, Lee A, Brimblecombe J (2021). Cultural adaptation of health interventions including a nutrition component in Indigenous peoples: a systematic scoping review. International Journal for Equity in Health.

[38] Mozaffarian D, Rosenberg I, Uauy R (2018). History of modern nutrition science-implications for current research, dietary guidelines, and food policy. BMJ.

[39] Kuhnlein HV, Erasmus B, Spigelski D, Burlingame B (2013). Indigenous peoples’ food systems and wellbeing: Interventions and policies for healthy communities.

[40] Browne J, Gilmore M, Lock M, Backholer K (2020). First Nations peoples’ participation in the development of population-wide food and nutrition policy in Australia: a political economy and cultural safety analysis. International Journal of Health Policy and Management.

[41] Elliott B, Jayatilaka D, Brown C, Varley L, Corbett KK (2012). “We are not being heard”: Aboriginal perspectives on traditional foods access and food security. Journal of Environmental and Public Health.

[42] Haraway DJ (1988). Situated knowledges: The science question in feminism and the privilege of partial perspective. Feminist Studies.

[43] Nazarea VD (1999). Ethnoecology: Situated knowledge/located lives.

[44] Carrasco E, Soto JL, Rojas W, Soto JL, Pinto M, Jäger M, Padulosi S (2010). Granos Andinos: avances, logros y experiencias desarrolladas en Quinua, Canihua y Amaranto en Bolivia.

[45] Repo-Carrasco R, Espinoza C, Jacobsen SE (2003). Nutritional value and use of the Andean crops quinoa (Chenopodium quinoa) and kañiwa (Chenopodium pallidicaule). Food Reviews International.

[46] Ayala G (2004). Seminario J, (ed.). Raíces Andinas: Contribuciones al conocimiento y a la capacitación.

[47] Argentina (1967). Ley 17259: La sal para uso alimentario humano o animal, deberá ser enriquecida con yodo.

[48] López Linares S, Martín Heer I (2014). Contenido de yodo en sal a nivel de puestos de venta provenientes de distintas localidades en tres regiones argentinas. Revista Argentina de Endocrinología y Metabolismo.

[49] Argentina (2002). Ley 25630: Anemias y malformaciones del tubo neural.

[50] Calvo EB, Biglieri A (2008). Impacto de la fortificación con ácido fólico sobre el estado nutricional en mujeres y la prevalencia de defectos del tubo neural. Archivos Argentinos de Pediatría.

[51] Rose G (2001). Sick individuals and sick populations. International Journal of Epidemiology.

